# Joint association between accelerometry-measured daily combination of time spent in physical activity, sedentary behaviour and sleep and all-cause mortality: a pooled analysis of six prospective cohorts using compositional analysis

**DOI:** 10.1136/bjsports-2020-102345

**Published:** 2021-05-18

**Authors:** Sebastien Chastin, Duncan McGregor, Javier Palarea-Albaladejo, Keith M Diaz, Maria Hagströmer, Pedro Curi Hallal, Vincent T van Hees, Steven Hooker, Virginia J Howard, I-Min Lee, Philip von Rosen, Séverine Sabia, Eric J Shiroma, Manasa S Yerramalla, Philippa Dall

**Affiliations:** 1 School of Health and Life Sciences, Glasgow Caledonian University, Glasgow, UK; 2 Department of Movement and Sports Sciences, Ghent University, Gent, Belgium; 3 Biomathematics and Statistics Scotland, Edinburgh, UK; 4 Department of Medicine, Columbia University Medical Center, New York, New York, USA; 5 Division of Physiotherapy, Department of Neurobiology, Care Sciences, and Society (NVS), Karolinska Institute, Stockholm, Sweden; 6 Department of Health Promoting Science, Sophiahemmet University College, Stockholm, Sweden; 7 Academic Primary Health Care Center, Stockholm, Region Stockholm, Sweden; 8 Federal University of Pelotas, Pelotas, Brazil; 9 Accelting, Almere, The Netherlands; 10 Exercise Science and Health Promotion Program, College of Health Solutions, Arizona State University, Phoenix, Arizona, USA; 11 University of Alabama School of Medicine, Birmingham, Alabama, USA; 12 Division of Preventive Medicine, Brigham and Women's Hospital, Harvard Medical School, Boston, Massachusetts, USA; 13 Inserm U1153, Epidemiology of Ageing and Neurodegenerative Diseases, Université de Paris, Paris, France; 14 Department of Epidemiology and Public Health, University College London, London, UK; 15 Laboratory of Epidemiology and Population Science, National Institute on Aging, Bethesda, Maryland, USA

**Keywords:** physical activity, sleep, health

## Abstract

**Objective:**

To examine the joint associations of daily time spent in different intensities of physical activity, sedentary behaviour and sleep with all-cause mortality.

**Methods:**

Federated pooled analysis of six prospective cohorts with device-measured time spent in different intensities of physical activity, sedentary behaviour and sleep following a standardised compositional Cox regression analysis.

**Participants:**

130 239 people from general population samples of adults (average age 54 years) from the UK, USA and Sweden.

**Main outcome:**

All-cause mortality (follow-up 4.3–14.5 years).

**Results:**

Studies using wrist and hip accelerometer provided statistically different results (I^2^=92.2%, Q-test p<0.001). There was no association between duration of sleep and all-cause mortality, HR=0.96 (95% CI 0.67 to 1.12). The proportion of time spent in moderate to vigorous physical activity was significantly associated with lower risk of all-cause mortality (HR=0.63 (95% CI 0.55 to 0.71) wrist; HR=0.93 (95% CI 0.87 to 0.98) hip). A significant association for the ratio of time spent in light physical activity and sedentary time was only found in hip accelerometer-based studies (HR=0.5, 95% CI 0.42 to 0.62). In studies based on hip accelerometer, the association between moderate to vigorous physical activity and mortality was modified by the balance of time spent in light physical activity and sedentary time.

**Conclusion:**

This federated analysis shows a joint dose–response association between the daily balance of time spent in physical activity of different intensities and sedentary behaviour with all-cause mortality, while sleep duration does not appear to be significant. The strongest association is with time spent in moderate to vigorous physical activity, but it is modified by the balance of time spent in light physical activity relative to sedentary behaviour.

## Introduction

Physical inactivity is associated with several chronic diseases,^1^ 3.9–5.3 million annual premature deaths globally^2 3^ and a $67.5 billion per year cost to healthcare systems worldwide.^4^ Few people actually meet the recommended levels of physical activity.^5^ During the day, individuals engage mainly in sleep, sedentary behaviour (SB)^6^ and light physical activity (LIPA) such as walking.^7^ The health benefits of daily moderate to vigorous physical activity (MVPA) are well established but increasing evidence suggests that sleep, SB and LIPA also have important consequences for health.^7–9^

Canada has recently issued the first integrated 24-hour public health guidelines for adults and older adults^10^ following the introduction of 24-hour movement guidelines for children in Canada, Australia and UK several years ago.^11^ These 24-hour guidelines combine recommendations on sleep, SB and physical activity but are mainly based on evidence derived from studying the impact of each of the behaviours independently.

There is a lack of evidence about the joint association between time spent in sleep, SB and physical activity with health outcomes.^12^ Consequently, the WHO 2020 guidelines focus on physical activity and SB only.^13^ These guidelines highlight that some of the key remaining questions are how the combination of time spent in each of these basic elements of daily routine impacts our health, whether this reduces or enhances the benefits of recommended daily MVPA and whether an optimum daily combination of time exists.^12 14^ In order to answer this question, it is important to account for the finite nature of a day, given that time is limited to 24 hours in a day. Time spent in one movement behaviour necessarily influences the time that remains to be spent in the others. Therefore, time spent in sleep, SB, LIPA and MVPA are codependent. Compositional data analysis has been advocated as a methodological approach that accounts for this fact.^15–18^

Compositional data analysis is a well-established branch of statistics that deals with multivariate data that forms part of a finite whole.^19^ Exposure or recommendations expressed as time spent in movement behaviours and sleep per day or per week are compositional in nature as they form parts of a total, for example, the 24-hour day, the waking day, or a week. Consequently, compositional methods are starting to be used in physical activity research and epidemiology. The advantage of applying compositional methods, beyond properly dealing with the relative nature of the data, is that they allow the adjustment of models for time spent in all movement behaviours and enable the quantification of the joint associations between daily movement behaviours and health. This could provide evidence to underpin robust integrated movement behaviour guidelines. Janssen *et al* have systematically reviewed compositional analysis studies examining associations between sleep, SB and physical activity with health outcomes in adults as part of the evidence review for the Canadian 24-hour guidelines.^20^ Of the eight studies included in the review only one was prospective, demonstrating an urgent research need for further prospective compositional analysis evidence.^21^

Therefore, this study aimed to assess the prospective association of different combinations of time spent daily in physical activity, SB and sleep with mortality using compositional analysis applied to data from large-scale cohorts with device-measured time spent in movement behaviours. More precisely, this study aimed to estimate the joint effects of movement behaviours (sleep, SB and physical activity) on risk of all-cause mortality. In causal compositional analysis terms this refers to estimating the relative causal effect of each movement behaviour conditional on the other movement behaviours.^18^

## Methods

### Design

**Figure 1:**
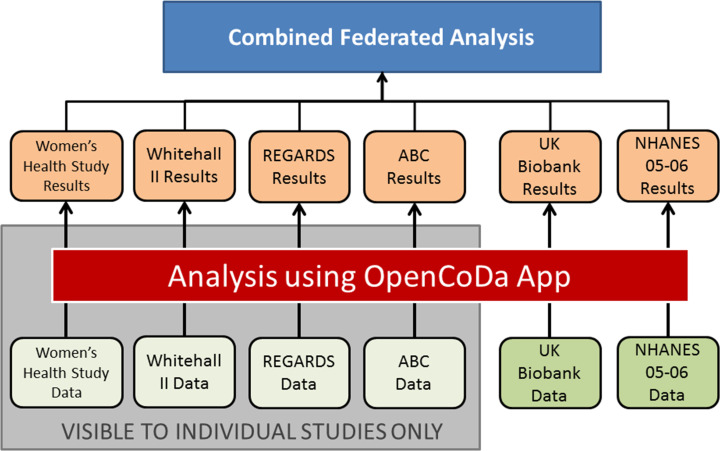
Structure of federated analysis employed. UK Biobank and National Health and Nutrition Examination Survey (NHANES) are open-source data sets.

This study adopted a federated pooled analysis approach to maximise the use of available data (figure 1).^22 23^ Under such an approach, an analytical methodology was created and automated, then distributed to individual studies to perform the analysis without the need to share data access across teams, avoiding any associated ethical and legal issues.

### Data sources and data set selection

To identify suitable data sets, we conducted a systematic search, following Preferred Reporting Items for Systematic Reviews and Meta-Analyses guidelines,^24^ of four databases (PubMed, PsycINFO, Embase and Web of Science) from database inception until 28 February 2018 (a detailed search description is provided in the online supplemental file). We included prospective cohort studies, reported in the English language, that had individual-level exposure and outcome data, provided data on time spent in SB, LIPA and MVPA measured using a body-worn sensor over 7 consecutive days, and all-cause mortality. We identified whether data from each of the studies were available in accessible, open-source databases. When data for a study were not publicly available we contacted an author or principal investigator on at least two occasions between March and October 2018 and invited them to participate.

10.1136/bjsports-2020-102345.supp1 Supplementary data

### Data analyses

We conducted the analysis according to Strengthening the Reporting of Observational Studies in Epidemiology guidelines.^25^ Authors with access to the individual-level data for each study analysed their data according to a standardised protocol implemented in R (R V.3.4.1, R Foundation for Statistical Computing, Vienna, Austria, 2017) and Shiny (Shiny V.1.0.5, RStudio, Boston, USA, 2017) that was made available through the OpenCoDa website (https://opencoda.net).

Each participating study examined the association between the daily composition of time spent in sleep, SB, LIPA and MVPA as exposure variables with all-cause mortality using a novel compositional Cox regression analysis.^26^ This method is an extension of standard Cox regression that enables computation of the association between the daily time composition, as an entire multicomponent exposure variable, with mortality (or any other time-to-event outcome variable) taking into account the codependence and interactions between behaviours making up the composition. The method is detailed below and R codes are available at www.opencoda.net.

In this study, the composition of the day was defined as the proportions of time spent in *D*=4 movement behaviours: *MVPA*, *LIPA*, *SB* and sleep (*Sleep*). How each cohort study measured the time spent in each behaviour is detailed in online supplemental table A1.

To be treated and interpreted correctly, information contained in parts of a composition needs to be expressed relative to the other parts as log ratios.^19^ Therefore, participant's times spent in sleep, SB, LIPA and MVPA were transformed into three isometric log ratio (ilr) coordinates^27^ given by equations 1–3:

(1) $$z_{1}=\sqrt{\frac{3}{4}}\ln\left(\frac{Sleep}{\sqrt[{3}]{MVPA\cdot LIPA\cdot SB}}\right)$$

(2) $$z_{2}=\sqrt{\frac{2}{3}}\ln\left(\frac{MVPA}{\sqrt{LIPA\cdot SB}}\right)$$

(3) $$z_{3}=\sqrt{\frac{1}{2}}\ln\left(\frac{LIPA}{SB}\right)$$

These fully represent the composition and guarantee desirable formal properties, such as orthonormality and consistence if working with a subcomposition regardless of the scale of measurement of the data or the total time period considered. This is important, for example, when using the waking day as a subcomposition, due to variation in the length of the waking day.^27^ The ilr coordinates devised represent nested contrasts of relative importance between subsets of behaviours. The first coordinate $z_{1}$ represents time spent in sleep relative to the (geometric) mean of all the other behaviours. The second coordinate $z_{2}$ is the balance between time allocated to MVPA and time allocated to (the geometric mean of) LIPA and SB. The third coordinate $z_{3}$ accounts for the balance of time between LIPA and SB. Note that it is possible to consider other ilr coordinate systems in the Cox regression model to investigate the significance of other time balances of interest.^26^ However, the estimated responses and global parameters from the model would be the same. Our choice of the balance between sleep time and the other behaviours as the first coordinate $z_{1}$ facilitated the pooling of studies whether they measured sleep or not (detailed below). This set constrains the influence of sleep time to a single coordinate (in view of the presence of studies that did not measure Sleep time).

These coordinates were then used as explanatory variables to fit a Cox regression model of the form:

(4) $$\ln\left(\frac{h\left(t;w\right)}{h_{0}\left(t\right)}\right)=\sum_{j=1}^{D-1}\gamma_{j}z_{j}+\beta^{T}v=\gamma^{T}z+\beta^{T}v$$

where $h_{0}\left(t\right)$ , depending on time t , is an unspecified baseline hazard function, the vectors $z={\left(z_{1},z_{2},z_{3}\right)}^{T}$ and v refer respectively to the ilr coordinates and any other covariates (with them all jointly forming the vector of explanatory variables $w={\left(z^{T},v^{T}\right)}^{T}$ ), and the vectors γ and β are the corresponding regression coefficients. These coefficients were fitted in the usual manner by maximising the partial likelihood function.^26^ The term h(t;w) is the ordinary hazard function, which relates to probability of survival to time t , S(t) , through equation 5:

(5) $$S\left(t\right)=\exp\left(-\int_{0}^{t}h\left(u\right)du\right)$$

The protocol provided a list of covariates to be included in the model based on the causal assumptions shown in online supplemental figure A1. These assumptions were informed by the compositional causal inference framework developed by Arnold *et al* 18 and the recent review in physical activity research.^28^ Each cohort selected the relevant data (detailed in online supplemental table A2) to represent these covariates: demographic information (age, sex and ethnicity), socioeconomic status, health status and/or pre-exiting conditions, health-related behaviours (smoking, diet and alcohol consumption). In order to address the lag in time between the measurement of the composition and covariates in the UK Biobank cohort we implemented the same methodology as Strain *et al* 29 to quantify the study covariates.

For each cohort, the estimated associations between the above time balances, as represented by the ilr coordinates, and mortality were expressed as HRs with their corresponding 95% CIs.^26^ In order to minimise the risk of reverse causation bias, we excluded deaths in the first 2 years of follow-up in each of the studies. The proportional hazards assumption was verified at the individual study level by examination of the Kaplan-Meier curves and a Grambsch-Therneau test.^30^

Because compositional data are expressed as log ratios, zero time recorded in a part of the composition creates a mathematical issue. Therefore, time budgets need to be preprocessed to deal with zeroes. For each study, any zeroes present in the compositional variables (Sleep,MVPA,LIPA,SB) were assumed to represent unobserved small values, for example, resulting from rounding off, falling below detection thresholds or limited observation time, and were imputed using the log ratio EM algorithm implemented for this purpose in the function lrEM of the R package *zCompositions*.^31^ Less than 2% of individuals had zero-valued *MVPA*. Detection limits were set by study based on the measurement and epoch used, for example, 1 min for accelerometer measurements of physical activity based on 1 min epochs.

### Pooling

Each individual study provided the estimated model coefficients and the variance-covariance matrix of these coefficients, which were used to obtain the pooled coefficients and SEs using the random effects model implemented in the R package *mvmeta*, whether a study measured sleep or not.^32^ As indicated, the ilr coordinates were chosen to restrict sleep to the first coordinate $z_{1}$ . To pool results, the coefficient for $z_{1}$ was set to zero in studies which did not measure sleep, and the variance was set to an arbitrarily high value. This ensured that the studies that did not measure sleep were not taken into account in the estimation of coefficients for $z_{1}$ . Their contribution to estimated coefficient for $z_{1}$ was thus made negligible in the pooled results.

The mortality HRs were estimated from the pooled model to ascertain the association between the daily four-behaviour time composition and all-cause mortality. The HR between any two compositions (say 1 and 2) expressed in ilr coordinates $z^{1}$ and $z^{2}$ respectively was calculated as

(6) $$HR=exp\left(\sum_{i=1}^{3}\left(\beta_{i}z_{i}^{2}-\beta_{i}z_{i}^{1}\right)\right)$$

To select a reference composition, we fitted a normal distribution to the observed second and third ilr coordinates, then computed a 75% confidence region based on a contour of constant Mahalanobis distance (a multivariate distance accounting for the covariance structure).^33^ We then selected a point on this outer contour and applied inverse ilr transformation to obtain a reasonable composition of low MVPA and LIPA and sedentary time but that still lay within a region where our model was well supported by the data (online supplemental figure A2).

### Heterogeneity and sensitivity analysis

We investigated heterogeneity between studies using the I^2^ statistics and Cochran’s Q-test. In order to ascertain the robustness of the results and to investigate potential sources of heterogeneity we repeated the pooled analysis with the following conditions: (A) we excluded one study in turn (leave-one-out procedure), (B) we excluded all studies using wrist-worn accelerometers, and (C) we excluded all studies using hip-worn accelerometers. We examined the difference in model coefficients for each ilr coordinate in each of these conditions.

## Results

The systematic search identified 12 studies as eligible.^34–46^ Of these, six did not respond to our request to participate^36 42–46^ by the deadline of September 2019. Data from two studies were available publicly^35 37^ and four studies agreed to participate.^34 39–41^ These six studies were included in the analysis (table 1).

**Table 1:** List characteristics of studies included in the federated analysis

Study	Country; sample; deaths	Year of baseline assessment; mean follow-up	Device; method of sleep time assessment	Method of death ascertainment	Covariates
ABC	Sweden; n=841 (370 men, 471 women); 78 deaths	2001–2002; 14.5 years	ActiGraph 7164 (lower back); NA	National death register	Age, sex, education level, alcohol consumption status, smoking status, self-assessed health
NHANES	USA; n=2927 (1453 men; 1474 women); 283 deaths	2003–2006; 6.7 years	ActiGraph 7164 (hip); NA	National Death Index	Age, sex, ratio of family income to poverty, alcohol consumption, smoking status, energy intake, self-assessed health, physical limitations on movement, existing diagnosed medical condition (coronary heart disease, stroke, cancer, diabetes), hypertension
REGARDS	USA; n=7076 (3257 men; 3819 women); 477 deaths	2003–2007; 5.7 years	Actical (hip); NA	Review of death certificates, medical records and administrative databases	Age, sex, BMI, education, race, region of residence, season accelerometer was worn, current smoking, alcohol use, diabetes, hypertension, dyslipidaemia, estimated glomerular filtration rate, atrial fibrillation, history of coronary heart disease, history of stroke, self-assessed health
UK Biobank	UK; n=98 819 (43 229 men; 55 590 women); 2411 deaths	2013–2015; 5.4 years	Axivity AX3 (dominant wrist); self-report	National Health Service central registers	Age, sex, BMI, education, race, Townsend deprivation index, alcohol consumption, smoking status, self-assessed health, fruit and vegetable consumption, oily fish consumption, salt intake, red meat consumption, use of blood pressure or cholesterol medicine, physical limitations on movement, existing medical condition (cardiovascular disease, stroke, cancer), existing diagnosis of diabetes (self-reported diagnosis or use of insulin)
Whitehall II	UK; n=3900 (2891 men; 1009 women); 140 deaths	2012–2013; 5.3 years	GENEActiv Original (non-dominant wrist); accelerometer	National Health Service central registers	Age, sex, occupational position, alcohol consumption, smoking status, fruit and vegetable consumption, health state (multimorbidity index made of history of diabetes, coronary heart disease, stroke, depression, cancers, arthritis, chronic obstructive pulmonary disease, dementia and Parkinson’s disease)
Women’s Health Study	USA; n=16 676 (all women); 503 deaths	2011–2015; 4.3 years	ActiGraph GT3X+ (hip); NA	Review of medical records, death certificates or the National Death Index	Age, sex, income, smoking status, alcohol consumption, saturated fat intake, fibre intake, fruit and vegetable consumption, hormone therapy, family history of myocardial infarction, family history of cancer, general health, history of cardiovascular disease, history of cancer, results of cancer screen

BMI, body mass index; NA, not applicable; NHANES, National Health and Nutrition Examination Survey.

These studies included 130 239 individuals who were followed on average from 4.3 to 14.5 years depending on the cohort, during which 3892 (2.98%) died. Two of the studies included data on sleep time assessed using wrist-worn devices^40^ and were entered in the analysis of the association of sleep time relative to other behaviours with all-cause mortality. Demographics and average (compositional centre) pattern of time in each behaviour for each study are presented in table 2.

**Table 2:** Demographics and average daily time composition (computed as compositional geometric means) per study

Study	% women	Average age (years)	MVPA (min/day)	LIPA (min/day)	SB (min/day)	Sleep (min/day)
ABC	44.0	52.8	23.9	379.8	556.3	NA
NHANES 2003–2006	49.3	63.6	8.2	355.5	596.3	NA
REGARDS	54.0	63.4	5.2	193.2	761.7	NA
UK Biobank	56.2	62.3	61.8	122.6	818.3	437.3
Whitehall II	25.9	69.4	46.9	93.5	835.8	463.7
Women’s Health Study	100.0	72.0	7.0	303.5	649.5	NA

LIPA, light physical activity; MVPA, moderate to vigorous physical activity; NA, not applicable; NHANES, National Health and Nutrition Examination Survey; SB, sedentary behaviour.

**Figure 2:**
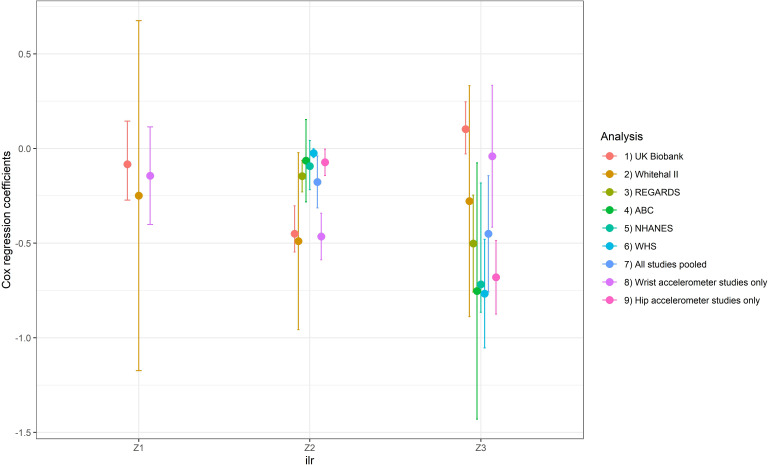
Results of the sensitivity analysis showing Cox regression model coefficients with 95% CI bars for each of the isometric log ratio (ilr) coordinates ( $z_{1}$ , $z_{2}$ , $z_{3}$ ) in models including all studies, leave-one-out models and in models pooling studies using wrist accelerometers only and hip accelerometers only. For hip accelerometers, only $z_{1}$ was not calculated. NHANES, National Health and Nutrition Examination Survey; WHS, Women’s Health Study.

Meta-analysis pooling results from all studies showed significant heterogeneity (I^2^=92.2%, Q-test p<0.001). The sensitivity analysis revealed that the most important source of heterogeneity arose from accelerometer placement. Figure 2 shows that the model coefficients were significantly different when results were pooled according to accelerometer body placement. This strongly suggests that pooling results from studies using wrist accelerometers together with those using hip-mounted accelerometers might not be appropriate. Consequently, we present below results stratified according to accelerometer body placement (table 3). These stratified analyses showed low to moderate heterogeneity which was not significant according to Q-test (I^2^=16.3%, p=0.31 for wrist accelerometer studies and I^2^=30.3%, p=0.20 for hip accelerometer studies). Hip-mounted studies only measured waking day behaviour, therefore results for the first ilr coordinate (which includes sleep) are not presented.

**Table 3:** Estimated coefficients for Cox regression model on isometric log ratio (ilr) coordinates of daily time composition pooled for studies using wrist accelerometers and studies using hip accelerometers, HRs, p values and 95% CIs

Log ratio	Coefficient	Unit HR*	P value†	95% CI lower bound	95% CI upper bound
Wrist accelerometer studies					
$z_{1}=\sqrt{\frac{3}{4}}\ln\left(\frac{Sleep}{\sqrt[{3}]{MVPA∙LIPA∙SB}}\right)$	−0.144	0.962	0.275	−0.402	0.114
$z_{2}=\sqrt{\frac{2}{3}}\ln\left(\frac{MVPA}{\sqrt{LIPA∙SB}}\right)$	−0.465	0.628	<0.001	−0.588	−0.342
$z_{3}=\sqrt{\frac{1}{2}}\ln\left(\frac{LIPA}{SB}\right)$	−0.041	0.960	0.829	−0.415	0.333
Hip-mounted accelerometer studies					
$z_{2}=\sqrt{\frac{2}{3}}\ln\left(\frac{MVPA}{\sqrt{LIPA∙SB}}\right)$	−0.073	0.930	0.040	−0.143	−0.003
$z_{3}=\sqrt{\frac{1}{2}}\ln\left(\frac{LIPA}{SB}\right)$	−0.681	0.506	<0.001	−0.875	−0.486

*This figure, calculated as exp(*β* coefficient), represents the HR in respect of an increase of 1 unit in the corresponding ilr coordinate. A value greater than 1 indicates that higher values of the behaviour balance are associated with increased mortality risk, and a value lower than 1 indicates that higher values of the behaviour balance are associated with decreased mortality risk.

†P values and 95% CIs are based on Wald test statistics.

### Pooled analysis using wrist accelerometers

**Figure 3:**
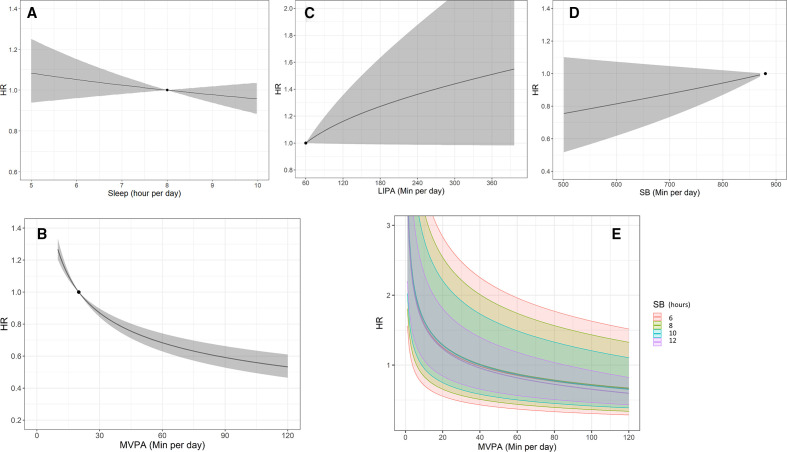
Results from pooled studies using wrist accelerometers. Dose–response relationship (with 95% CI—ribbons) between time spent in (A) sleep (relative to all other behaviours), (B) moderate to vigorous physical activity (MVPA) (relative to all other behaviours), (C) light physical activity (LIPA) (relative to all other behaviours), (D) sedentary behaviour (SB) (relative to all other behaviours), (E) joint association between time in MVPA, SB (presented as different levels) and LIPA (implied as it makes up the remaining time in the waking day time in LIPA=16 hours−time in MVPA−time in SB). HRs were computed with respect to the following reference composition defined as described in the Methods section: MVPA=20 min/day, LIPA=60 min/day, SB=14 hours and 40 min/day, sleep=8 hours/day (marked as a solid black dot). Compositions with different values in time in the primary behaviour were reported such that the remaining behaviours were in the same ratio as for the reference composition.

The proportion of time spent sleeping relative to the other behaviours was not significantly associated with all-cause mortality (table 3, $z_{1}$ ). This is illustrated in figure 3A showing that the dose–response relationship between the risk of all-cause mortality and sleep time is nearly flat. For the waking day behaviours, there was a statistically significant association between the time spent in MVPA compared with other waking day behaviours ( $z_{2}$ ) and all-cause mortality. MVPA had a curvilinear dose–response relationship with lower risk of all-cause mortality at higher time spent in MVPA (figure 3B). The third ilr coordinate ( $z_{3}$ ), involving the ratio of time spent in LIPA and SB, was not statistically significant in this analysis (table 3), hinting that the balance of time between SB and LIPA is not associated with all-cause mortality. The dose–response curves for LIPA and sedentary time are shown in figure 3C, D. While these show trends towards higher risk with higher time spent in either of these behaviours, they are not statistically significant. The joint association between the waking day behaviours and all-cause mortality is presented in figure 3E. This curve shows that the association between MVPA and all-cause mortality was not significantly modified by the balance of time spent in LIPA or SB. In figure 3E, the similarity of the dose–response curves of MVPA across a range of values of sedentary time (and thus also across a range of different values of time spent in LIPA as it constitutes the remaining time of the waking day) shows that the relationship was not significantly modified by time spent in LIPA.

### Pooled analysis using hip accelerometers

**Figure 4:**
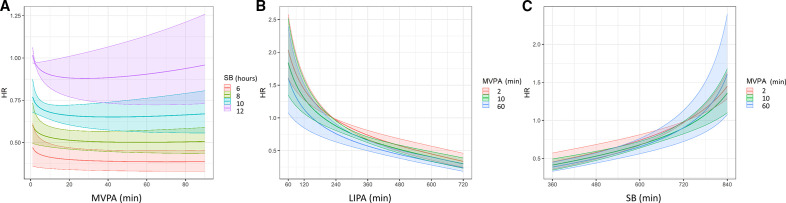
Results from pooled studies using hip accelerometers. Dose–response relationship (with 95% CI—ribbons) for waking day behaviours between time spent in (A) moderate to vigorous physical activity (MVPA) for different levels of sedentary time (with light physical activity (LIPA) making up the remaining time in the waking day), (B) LIPA for different levels of MVPA (with sedentary time making up the remaining time in the waking day), and (C) sedentary behaviour (SB) for different levels of MVPA (with LIPA making up the remaining time in the waking day). HRs were computed with respect to the following reference composition defined as described in the Methods section: MVPA=2 min/day, LIPA=229 min/day, SB=729 min/day, sleep=8 hours/day.

For pooled studies using hip accelerometers, the analysis also revealed a statistically significant association between the proportion of time spent in MVPA compared with other waking day behaviours ( $z_{2}$ ) and all-cause mortality. Additionally, there was a statistically significant association between the proportion of time spent in LIPA compared with sedentary time ( $z_{3}$ ) and all-cause mortality (table 3). MVPA had a curvilinear dose–response relationship with lower risk of all-cause mortality at higher time spent in MVPA relative to other waking day behaviours which tended to flatten after around 20 min/day (figure 4A). However, the benefits are lower at higher levels of sedentary time (and therefore lower levels of LIPA as it is the remaining time of the waking day) and fully attenuated for daily sedentary time exceeding 11–12 hours/day. Time spent in LIPA shows a curvilinear dose–response with lower risk of all-cause mortality at higher levels of LIPA (figure 4B) that is not significantly modified by the time spent in MVPA. In contrast, higher sedentary time is associated with higher mortality risk (figure 4C) and this is not significantly modified by the time spent in MVPA.

**Figure 5:**
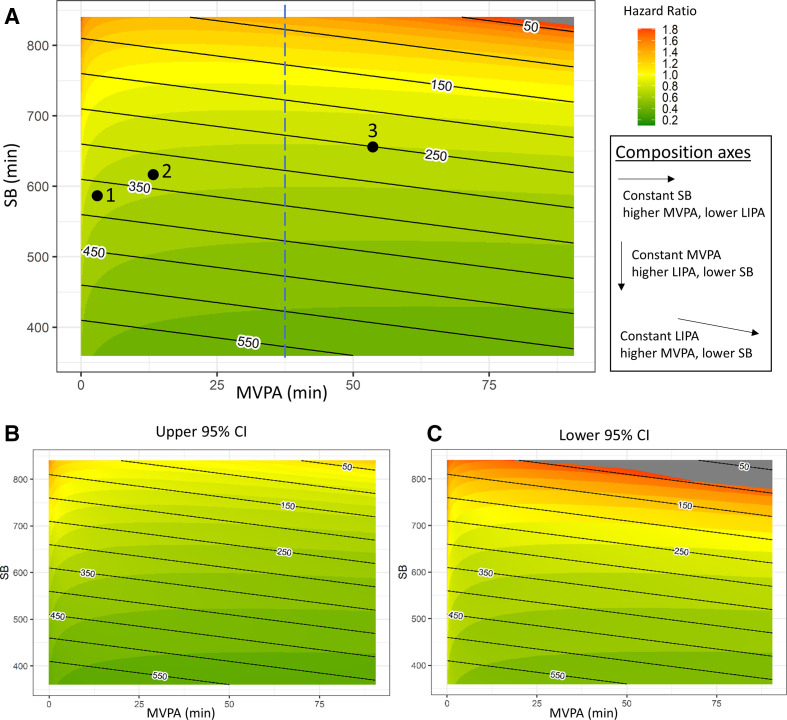
Results from pooled studies using hip accelerometers. HRs for different compositions of the waking day are presented as a heat map in (A). Moderate to vigorous physical activity (MVPA) and sedentary time are shown on the x-axis and y-axis, respectively. The remaining time in the waking day is made up of light physical activity (LIPA), black lines represent LIPA isotime lines. The dashed blue line represents composition with 30 min of MVPA. Heat maps of the lower and upper bounds of the 95% CIs are shown in (B) and (C). HRs were computed with respect to the following composition: MVPA=2 min/day, LIPA=229 min/day, SB=729 min/day, sleep=8 hours/day. SB, sedentary behaviour.

Heat maps (figure 5) show the joint associations between time spent in MVPA, LIPA and SB with all-cause mortality in more detail. HRs for waking day behaviour are shown against MVPA and SB. For each data point in the heat maps, the remaining time in the waking day is made up of LIPA, and LIPA isotime lines have been added to the graph for ease of interpretation. Heat maps of the upper and lower bounds of the 95% CIs are provided in figure 5B, C. MVPA does not appear to be the only behaviour that affects risk. For example, spending 30 min in MVPA is associated with a wide range of HR from 1.6 to 0.2, depending on how the remaining waking time is divided across SB and LIPA (figure 5A, blue dashed line). A range of different combinations of time spent in waking day behaviours are associated with similar lower risk of all-cause mortality. For example, point 1 (MVPA=3 min/day, LIPA=375 min/day, SB=582 min/day), point 2 (MVPA=13 min/day, LIPA=330 min/day, SB=617 min/day) and point 3 (MVPA=55 min/day, LIPA=250 min/day, SB=655 min/day) in figure 5A all correspond to HR=0.70 (95% CI 0.65 to 0.75). How the dose–response between MVPAs is modified by other waking day behaviours is shown in figure 5A. Generally, compositions with higher MVPA and LIPA and lower sedentary time are associated with lower risk of all-cause mortality. This suggests that displacing sedentary time with physical activity of any intensity is beneficial. Table 4 shows differences in compositions associated with a 10% lower risk of all-cause mortality compared with three reference compositions. At low levels of MVPA (eg, 2 min/day), displacing sedentary time with MVPA appears to be six times more time efficient compared with displacing sedentary time with LIPA. Above 30 min/day of moderate-to-vigorous physical activity, displacing sedentary time with LIPA was just as time efficient as displacing sedentary time with MVPA.

**Table 4:** Estimated time difference in waking day composition associated with a risk reduction of 10% in all-cause mortality (HR=0.90) with respect to the reference composition

Reference composition	MVPA=2 min/day LIPA=358 min/day SB=10 hours/day	MVPA=10 min/day LIPA=350 min/day SB=10 hours/day	MVPA=30 min/day LIPA=330 min/day SB=10 hours/day
Composition difference			
More MVPA and less SB (LIPA is fixed)	8 min (95% CI 3 to 91)	29 min (95% CI 13 to 95)	52 min (95% CI 31 to 98)
More LIPA and less SB (MVPA is fixed)	51 min (95% CI 39 to 79)	50 min (95% CI 39 to 72)	49 min (95% CI 37 to 70)

Computations are based on hip accelerometer data.

LIPA, light physical activity; MVPA, moderate to vigorous physical activity; SB, sedentary behaviour.

## Discussion

This large federated analysis, comprising 130 239 participants across six prospective cohort studies, provides important data about the joint associations between daily movement behaviours and health that could inform public health recommendations and interventions targeting movement behaviours.

We observed a curvilinear dose response between time spent in MVPA and risk of all-cause mortality, with lower risk associated with higher time spent in MVPA. The shape of the relationship suggests that there are diminishing returns in engaging in MVPA. Most of the benefits are seen below 30 min of daily MVPA. This supports the large existing body of research showing the beneficial effect of physical activity.^2 47^

However, there was a clear heterogeneity of results between studies using wrist- and hip-mounted accelerometers. Studies using a wrist accelerometer tended to result in stronger association for MVPA and suggest that it is only time spent in this behaviour relative to the others that is associated with all-cause mortality. On the contrary, studies using hip accelerometers showed a beneficial association between LIPA and all-cause mortality, which supports recent suggestions that LIPA could be beneficial for health^7 8 48^ and a detrimental association of time spent in SB with all-cause mortality which is consistent with a growing body of evidence about the detrimental effect of SB on health.^9^ These results are in good agreement with a recent similar study^48^ and add new evidence about joint associations. Notably, we show that the beneficial association of time spent in MVPA with all-cause mortality depends on the balance of time spent in LIPA and SB. At high sedentary time (over 11–12 hours) and therefore lower time spent in LIPA, the benefits of MVPA might be completely attenuated. This is in contradiction with reports based on self-reported data^49^ but in good agreement with more recent studies based on accelerometer data.^50^

Our results suggest that there might be different behavioural pathways to achieve health benefits through encouraging the displacement of sedentary time by either MVPA or LIPA. Interventions, guidelines or policies advocating increases in LIPA and decreases in SB could be useful in reducing the health burden of inactivity, considering how few people achieve the recommended guidelines for MVPA.^51^ This could open the door to interventions and recommendations tailored to individual circumstances and capacity. LIPA could be important for people who cannot engage in MVPA, although this is potentially between two and six times less efficient in terms of time investment (see table 4). On the other end of the spectrum, LIPA appears just as beneficial as MVPA for people who engage in over 30 min/day in MVPA.

This difference in results between wrist-worn and hip-worn accelerometers is not entirely surprising as differences in accelerometer placement have been hotly debated to find the right balance between accuracy and increasing sample size through better compliance to measurement protocols. It asks the question about which of these results are the most likely. In this study all the cohorts used threshold-based methods to classify time spent in the different behaviours. Recent validation studies show that threshold methods for both hip and wrist accelerometers provide valid estimates of sleep time.^52^ On the contrary, poor correlation between wrist and hip accelerometer count-based metrics has been widely reported.^53 54^ The validation studies tend to find that hip accelerometers provide more accurate estimates than wrist accelerometer based on count threshold classification methods.^55^ Particularly, threshold-based classification of wrist accelerometer data leads to overestimation of time spent in MVPA and a large misclassification between SB and LIPA.^56 57^ These misclassifications of sedentary time as LIPA and overestimation of time spent in MVPA are likely to significantly attenuate the association for LIPA and SB in the results of the wrist accelerometer studies. Despite larger sample sizes in the studies using wrist accelerometers, the results of the hip accelerometer studies are at face value more likely to reflect the relationships for waking day behaviours. This might not be the case if other methods of behaviour classification are used. Indeed, wrist accelerometers are more likely to assess sporadic movement in addition to physical activities, particularly with a resolution of the acceleration signal at 5 s. Although these movements generate energy expenditure, their benefit for health might not be similar to those from sustained activities. Further studies should investigate the importance of bout duration in wrist accelerometer data for inference in health research.

We did not find a clear indication that sleep time was associated with all-cause mortality. This is, however, based on only two studies and a narrow range of sleep time recorded in these studies. In contrast, several studies report a dose–response between sleep and mortality, although the shape of the dose–response is contested.^8^ A possible explanation is that the observed association in previous research could be due to the lack of adjustment for waking day behaviour which is accounted for in our analysis. It is also important to note that a variety of measurement techniques were employed to measure sleep time across the various studies. In addition, sleep time is not regarded as a good indicator of healthy sleep behaviour,^58^ so our results do not preclude other dimensions of sleep being important for health.

Overall, our study suggests that future research should further investigate the relationship between the balance of time spent daily in these behaviours and health using different methods of accelerometer data processing to harmonise hip and wrist accelerometer data and/or use coordinated pooling of postural data.^59 60^ In particular, further prospective studies are required to provide evidence to underpin 24-hour movement behaviour recommendations.

Our analysis has several strengths. First, the use of accelerometers avoids the well-known pitfalls of self-report data. However, it should be noted that neither hip nor wrist accelerometers quantify actual sedentary time according to its established definition as time spent below 1.5 metabolic equivalents in a sitting or reclining posture^6^ as they do not measure posture. The large sample size allowed for a combined analysis of the dose–response associations among SB, physical activity and mortality that provided precise estimates with relatively narrow CIs. Mortality ascertainment varied across studies, but all used official national or regional registers which are likely to reflect a complete and accurate record. Lastly, and perhaps most importantly, the utilisation of a compositional data analysis approach ensures that the interplay between the different behaviours is properly accounted for.

The study has also some limitations. First, all of the studies were conducted in the USA and Western Europe and our search was limited to articles published in English. Thus, the results may not be generalisable beyond these populations. In addition, our search was systematic but not exhaustive of all possible databases, so it is possible that we have missed some data sources. However, we have checked the results of our search against the results of the exhaustive search conducted for the American physical activity guidelines.^1^ Second, residual confounding may exist. Although we aimed for broadly consistent covariates, differences exist between the studies, and difference in measurement of confounders might have distorted our results. We did not account for all potential confounders in our analysis, for example, genetic factors, environmental factors, body mass index and fitness were not considered (online supplemental material S1). Third, we attempted to minimise bias from reverse causation (ie, illness causing individuals to become sedentary) by restricting our analysis to free-living individuals, adjusting for the subject’s state of health at outset and by excluding death within the first 2 years in sensitivity analysis. However, we cannot fully rule out this bias.^61^ It is possible that ill individuals are more likely to die prematurely, as a consequence of engaging in less activity and more SB and sleep. This could result in overestimation of the association, but a recent study showed that this is unlikely to lead to entirely spurious associations.^62^

Our analysis did not examine specifically time spent in vigorous physical activity which was considered in a single category of movement behaviour together with moderate physical activity. Yet, recent research hints that the proportion of time spent in vigorous physical activity might be important for reducing mortality risk.^29 63^ Future research should consider daily time compositions with time spent in vigorous physical activity to understand the joint association between time spent in vigorous physical activity and other movement behaviours with mortality risk.

In conclusion, this federated analysis indicates that the daily balance of time spent in waking day behaviour physical activity of different intensity and SB shows a joint dose–response association with all-cause mortality. In general, combinations of time with lower SB and higher physical activity (of any intensity) are associated with lower all-cause mortality risk. The strongest association is with time spent in MVPA which should remain the focus of interventions and policy. However, our results also suggest that a number of different approaches could be taken towards health promotion in different populations. To reduce the risk of premature mortality, avoiding very long sedentary time and displacing SB with LIPA could also be a suitable strategy.

**What are the findings?**

- In this study, sleep time was not associated with all-cause mortality.
- Wrist and hip accelerometer data when processed using threshold classification methods lead to different results.
- Several different combinations of time spent in physical activities, sedentary behaviours and sleep are associated with a similar lower mortality risk.
- Replacing sedentary time with light physical activity provides health benefits but increasing moderate to vigorous physical activity requires less time for similar benefits.

**How might it impact on clinical practice in the future?**

- The results provide evidence for the development of integrated 24-hour movement guidelines.
- The results suggest that combined and flexible public health recommendations, policy and interventions tailored to an individual’s circumstances and capacities could be adopted. Future guidelines could be expressed in terms of a precise healthy balance of time spent in different waking day behaviours.
- As sedentary behaviour is so prevalent and most people are constrained to remain sedentary during the day at work, for example, it would allow them to match their activity levels to their levels of sedentary behaviour.

## Data Availability

Data are available in a public, open-access repository. Data may be obtained from a third party and are not publicly available. Some of the data sets included in this study are open access and others are curated by the studies. This is detailed in the manuscript and supplemental material.
