# Supplementary material for: Joint association between accelerometry-measured daily combination of time spent in physical activity, sedentary behaviour and sleep and all-cause mortality: a pooled analysis of six prospective cohorts using compositional analysis
Source: Br J Sports Med. 2021 May 18;55(22):1277–85. doi: 10.1136/bjsports-2020-102345 (PMC8543228; doi:10.1136/bjsports-2020-102345)
Supplement: Supplementary data [file bjsports-2020-102345supp001.pdf]

Supplementary material S1: Methodological details

Literature Search

One author (DM) searched four databases (PubMed, PsycINFO, Embase and Web of Science) from database inception to 28 February 2018 using the following search terms:
""sedentary"" OR ""sedentari"" OR ""sedentary behavio*"" OR ""sedentary lifestyle"" OR ""sedentary"" OR ""sitting"" OR ""sitting time"" OR ""physical inactivity"" OR ""inactivity*"" OR ""physically inactive""*
AND ""physical activity"" OR ""physical activities"" OR ""physically active"" OR ""physical exercise"" OR ""exercise"" OR ""walk*""*
AND ""mortality"" OR ""mortalities"" OR ""death"" OR ""fatal""

The systematic search retrieved 1,903 articles of which 149 papers for full text were screened, and 12 studies were identified as eligible for inclusion based on content of the paper and personal knowledge of the studies. The list of 12 was reviewed and confirmed by a second co-author (SC). Of these studies, six studies did not respond to our request or declined to participate (due to lack of time and resources), two studies' data was publicly available, and 4 studies agreed to participate. These 6 studies were included in the pooled analysis (Table 1). One further study suitable for inclusion was discovered to have been omitted after the conclusion of the federated analysis.

Accelerometry processing

Accelerometer data were processed by each local study with inclusion as per Table A4. Codes for processing are available on request or can be found on GitHub https://github.com/wadpac/code-published-studies/tree/master/WH2_McGregor

Accelerometer Thresholds

A variety of devices and protocols were used in the individual studies. Details of these are provided in Table A1.

Study	Device (location)	Data Used	Threshold for inclusion of individual	SB	LIPA	MVPA
ABC	Actigraph 7164 (lower back)	Uniaxial; 1 minute epoch	At least one valid day with 10+ hours of wear time	0 – 99 cpm	100 - 2019 cpm	2020+ cpm
NHANES 2003-06	Actigraph 7164 (right hip)	Uniaxial; 1 minute epoch	At least one valid day with 10+ hours of wear time	0 – 99 cpm	100 - 2019 cpm	2020+ cpm
REGARDS	Actical (right hip)	Uniaxial; 1 minute epoch	At least four valid days with 10+ hours of wear time / day	0 – 49 cpm	50 - 1065 cpm	1065+ cpm
UK Biobank	Axivity AX3 (dominant wrist)	Triaxial; 5 seconds epoch	At least 72 hours total wear time and wear data in each one-hour period of the 24-hour cycle	0 – 39 mg	40 – 99 mg	100+ mg

Whitehall II	GENEActiv Original (non-dominant wrist)	Triaxial; Bout based algorithm based on 5 seconds epoch	Daily wear time ≥2/3 of waking hours, for at least 2 weekdays and 2 week-end days.	0 – 39 mg	40 – 99 mg	100+ mg
Women's Health Study	ActiGraph GT3X+ (hip - unspecified)	Triaxial; 1 minute epoch	At least four valid day with 10+ hours of wear time / day	0 – 199 cpm	200 – 2689 cpm	2690+ cpm

Table A1 Details of individual study's physical activity data collection protocols.

Causal Assumptions

Selection of these covariates was based on the causal assumptions represented as a directed acyclic graph (DAG) below (Figure S1).

Our analysis was guided by the work of Arnold et al [1] on causal inference for compositional data and the recent review about the causal link between physical activity and longevity by Kujala [2]. Arnold et al have introduced DAG notation which are specific to causal inference for compositional data and their interpretation. In a DAG compositional variables are represented in a dashed box indicating that those variables occur at an instantaneous point in time. The composition, here the movement behaviour time composition, is fully determined by the sum of the parts. The deterministic relationship between variables representing the parts and the composition are indicated by double-lined arrow. In the case of compositional data, two distinct causal effect estimation need to be considered the total ('unconditional') effect of the composition on the outcome and the relative ('collider-conditional') effect. The latter is indicated in the DAG by dashed arrows and represents a joint effect of the balance between parts. For compositional data with fixed totals, which is the case of movement behaviours as the day is constrained to 24 hours, only the relative joint effect can be identified.

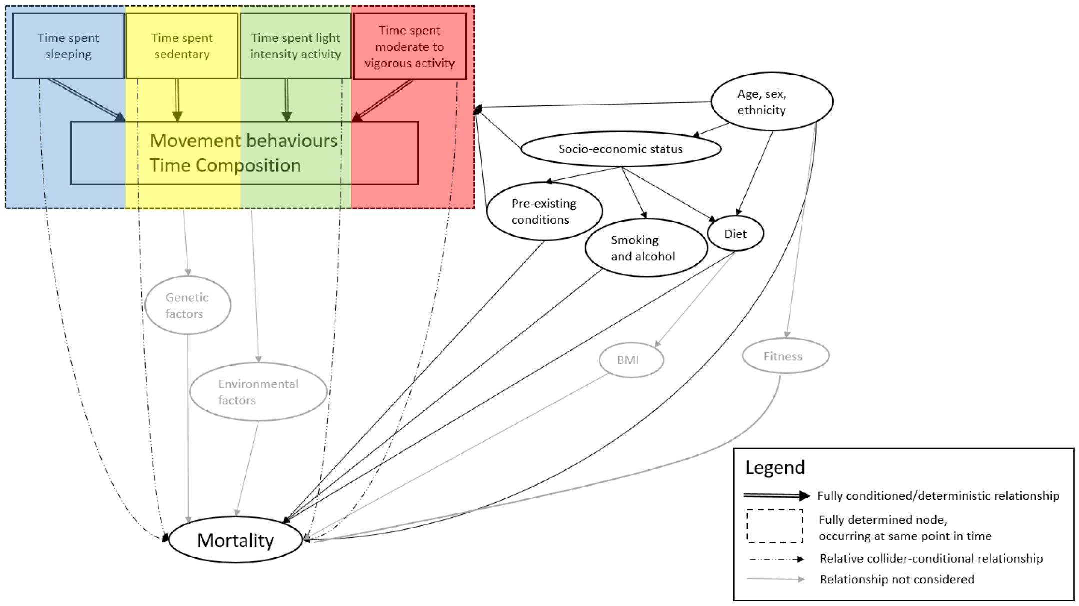

Figure A1: Directed acyclic graph illustrating the causal assumption of this study. Some relationships between factors have been omitted for clarity.

Summary results

The raw coefficients from the compositional Cox regression model are set out by study in Table A2. The sign of the coefficient indicates the sign of the association of the mortality outcome with the ilr-coordinate. "NA" indicates that sleep time was not available in the study.

Figure A2 below shows the compositional centres and density contour of compositions within the included studies. This shows the data coverage and the dispersion of compositions. Two clear cluster exist representing wrist and hip accelerometer data.

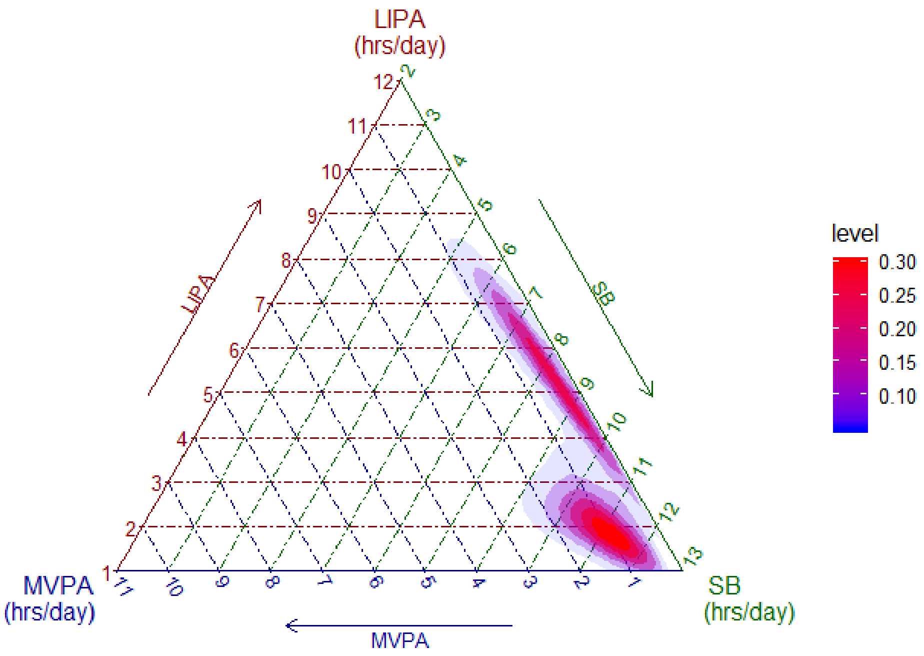

Figure A2: Density plot of waking day compositions, re-weighted to give equal probability weight to each study based on NHANES and UK Biobank data supplemented with synthetic data simulated based on summary statistics of the remaining studies and the additive logistic normal distribution.

Study	z_1	z_2	z_3
REGARDS	NA	-0.1461*	-0.5021*
ABC	NA	-0.0645	-0.7527*
Whitehall II	-0.2488	-0.4894*	-0.2779
UK Biobank	-0.0643	-0.4248*	0.1091
NHANES 03-06	NA	-0.0877	-0.5238*
Woman's Health Study	NA	-0.0244*	-0.7667*

* Statistically significant at 5% significance level based on Wald test statistic.

Table A2: Summary Cox regression coefficient estimates by individual study.
